# The Role of Stereotactic Body Radiotherapy (SBRT) in Oligoprogressive Renal Cell Carcinoma (RCC) Treated with ICIs–TKIs: A Retrospective Multicentric Study

**DOI:** 10.3390/jpm14101030

**Published:** 2024-09-27

**Authors:** Maria La Vecchia, Manuela Federico, Dario Aiello, Valentina Zagardo, Antonella Mazzonello, Lorella Testa, Leonarda La Paglia, Tiziana Bruno, Ivan Fazio

**Affiliations:** 1 Unità Operativa di Radioterapia Oncologica, Casa di Cura Macchiarella, 90138 Palermo, Italy; lavecchiamaria8@gmail.com (M.L.V.); dario_aiello@msn.com (D.A.); antonellamazzonella@alice.it (A.M.); lorellatesta@tiscali.it (L.T.); dina.lapaglia@gmail.com (L.L.P.); brtiziana@libero.it (T.B.); ivanfazio27@gmail.com (I.F.); 2 Unità Operativa di Radioterapia Oncologica, Rem Radioterapia srl, 95029 Viagrande, Italy; valentina.zagardo@gmail.com

**Keywords:** renal cell carcinoma, immunotherapy, stereotactic body radiotherapy

## Abstract

Background: This multicentric, retrospective study investigated the use of stereotactic body radiotherapy (SBRT) in patients (pts) with metastatic renal cell carcinoma (mRCC) who experienced oligoprogression during a combination therapy with an immune checkpoint inhibitor (ICI) and a tyrosine–kinase inhibitor (TKI). Methods: We retrospectively evaluated 34 pts affected by oligoprogressive RCC treated with an ICI–TKI combination between January 2020 and December 2023. SBRT was delivered to each site of oligoprogressive metastatic disease. After SBRT, pts were given follow-up clinical evaluations. 6–12–18-month local control (LC) rates and median next-line treatment-free survival (NEST-FS) were the primary endpoints. The secondary endpoints were overall response rate (ORR), clinical benefits and safety. Results: After a median follow-up of 24 months, 6–12–18-month LC rates were 100%, 71% and 43%, respectively, and the median NEST-FS was 20 months. ORR was 90%, while clinical benefit was 100%. No > G2 adverse events related to SBRT were recorded. Conclusions: In our study, SBRT for oligoprogressive mRCC turned out to be a safe and useful treatment which was able to preserve current treatment. Further prospective studies are necessary to explore the effects of the ICIs–TKIs combination and SBRT upon oligoprogressive sites in mRCC.

## 1. Introduction

Renal cell carcinoma (RCC) represents about 2.4% of all cancers in adults, with more than 400,000 new cases diagnosed worldwide per year [1].

The 5-year survival rate for kidney cancer for all patients (pts) is approximately 71%.

Survival rates strongly depend on disease stage, declining to 67% when locoregional disease (stage III) is detected and 12% in pts with distant metastases [2,3].

About 25–30% of pts with locally advanced disease present synchronous metastases and a proportion between 15 and 25% will develop metastases after nephrectomy [4,5].

The standard of care for pts with metastatic RCC (mRCC) is systemic therapy [3].

At present, therapeutic options for first-line systemic treatment of mRCC include the following:

- combination therapies with an immune checkpoint inhibitor (ICI) and a tyrosine–kinase inhibitor (TKI);
- combination therapy with two ICIs;
- monotherapy with a TKI.

Combination therapies using ICIs–TKIs and ICI–ICI were shown to be more effective than monotherapy with a TKI in intermediate and poor-risk (according to IMDC criteria) mRCC.

Particularly, KEYNOTE426, Checkmate 9ER and Clear trials explored the use of axitinib–pembrolizumab, cabozantinib–nivolumab and lenvatinib–pembrolizumab combinations, respectively, showing they are more effective than monotherapy with sunitinib [6,7].

The Checkmate214 trial, instead, compared a ipilimumab–nivolumab combination to monotherapy with sunitinib, showing longer median progression-free survival (PFS) and overall survival (OS) for the ICI–ICI combination [7].

Unfortunately, even during these very effective systemic treatments, resistances to drugs are developed [8].

Resistance to systemic therapy manifests as generalized or oligometastatic (OM) disease progression. The OM state is an intermediate state that may benefit from local treatments, such as surgery or radiotherapy (RT) [9].

Through the use of stereotactic body radiotherapy technique (SBRT), which delivers high doses in small volumes, the radioresistance of RCC has been overcome. Moreover, SBRT may activate the immune system against tumor cells, improving the effect of immunotherapy and delaying the change of systemic treatment [10,11].

Evidence has shown that SBRT used to treat OM disease guarantees a good local control (LC) with a tolerable toxicity in the management of many tumors [12] and in the treatment of associated tumor symptoms; for example, in the case of a tumor thrombus in the inferior vena cava, it can shrink edema and improve pts’ quality of life [13].

SABR-COMET is the first phase II randomized trial that assessed the impact of SBRT in patients with oligometastases belonging to multiple histologies. According to lesion location, which is the main cause of significant differences in LC rates, the overall long-term LC was better in patients who underwent SBRT [14].

Moreover, a long-term analysis of SABR-COMET showed improvements in OS with a median benefit of 22 months after a 5-year follow-up [14].

Therefore, even in mRCC, by using this strategy and combining RT with ICIs-based systemic treatments, LC may increase [9]. This study explores the role of SBRT in oligoprogressive mRCC treated with a first-line ICIs–TKIs combination.

## 2. Materials and Methods

We retrospectively evaluated 34 oligoprogressive mRCC pts and 56 metastases treated with ablative SBRT at the Department of Radiation Oncology of the Casa di Cura Macchiarella (Palermo, Italy) and at REM Department of IOM (Viagrande, Italy) between January 2020 and December 2023.

All pts signed an informed consent form before undergoing SBRT.

The indication for SBRT was given according to the following criteria: (1) Performance Status 0–2, (2) histologically proven primary RCC, (3) oligoprogressive lesion(s) during an ICIs–TKIs combination (<5 metastases), (4) oligoprogression detected after at least 6 months from the beginning of ICIs–TKIs treatment, and (5) almost 6 months follow-up. Pts receiving conventional fractionated radiation therapy, with the number of lesions at >5, without histologically proven primary RCC, were excluded. Patients with brain mts were excluded. Enrolled pts undergoing SBRT were treated with a dose of at least 6 Gy per fraction to a biologically effective dose (BED) of at least 90 Gy, using an α/β ratio of 3 Gy. Pts underwent computed tomography (CT)-based SBRT planning with a 3–5 mm slice thickness. Suitable immobilization devices were used, according to the site of the lesion. Diagnostic CT and MRI were co-registered with the planning CT to more accurately identify the target. Gross tumor volume (GTV), defined by means of morphologic and/or metabolic diagnostic instruments, was equivalent to the clinical target volume (CTV). The planning target volume (PTV) was defined by adding an isotropic 3–5 mm margin to the CTV. Organs at risk were delineated depending on the tumor site. The dose prescription depended on the volume and localization of oligometastases (OM). SBRT was delivered by Linac-based external beam radiation with daily Image-Guided Radiotherapy (IGRT). Before each treatment, image guidance with kilovoltage (kV) cone beam CT scans were acquired and appropriate adjustments were made to correlate bony anatomy. In lung treatment, breath control was performed with lung protocols cone beam CT and respiratory gating. During SBRT, TKIs were interrupted. All pts underwent consecutive daily sessions from Monday to Friday, and no discontinuity of treatment was observed.

After SBRT, pts were radiologically evaluated with CTCT or magnetic resonance imaging (MRI), depending on baseline imaging. The LC of each lesion was assessed according to Response Evaluation Criteria from the Solid Tumors RECIST criteria. Disease progression at >5 metastatic sites led to a change of systemic treatment. If further oligoprogression occurred, a new course of SBRT was proposed if less than 5 new metastases were diagnosed. SBRT retreatment was not allowed.

### Statistical Analysis

The primary endpoints were 6–12–18-month LC rates, defined as complete response (CR) and partial response (PR) of any duration or stable disease (SD) for at least 6 months from the start of RT in oligoprogressive sites. As coprimary endpoint, we chose next-line treatment-free survival (NEST-FS), defined as the time from the beginning of first-line systemic treatment to the change of systemic treatment. NEST-FS was first investigated and used as an endpoint in clinical trials exploring the use of metastases-directed therapies in other tumors, like prostate cancer, but has never been used in kidney cancer. However, it seems to be the most suitable endpoint to evaluate the role of metastases-directed therapies in oligoprogressive tumor disease. The secondary endpoints were overall response rate (ORR), defined as CR or PR of any duration after the start of RT, clinical benefits and safety.

## 3. Results

### 3.1. Patients Characteristics

A total of 34 pts with 56 oligoprogressive lesions receiving SBRT were retrospectively analyzed. The median age was 61 years (range 44–82). We evaluated 4 females and 30 males; 20, 10 and 4 pts had an ECOG PS of 0, 1 and 2, respectively. Pts’clinical characteristics are listed in Table 1. For mRCC, all pts received a first-line ICIs–TKIs combination. Particularly, 20 pts received a combination of axitinib–pembrolizumab, while 14 pts received cabozantinib–nivolumab. The best response to an ICIs–TKIs combination before SBRT was CR in 7, PR in 12 and SD in 15 pts. Imaging techniques used to detect oligoprogressive sites and evaluate responses to MDT were CT in 30 pts and MRI in 14 pts.

SBRT was delivered to 14 bone, 16 nodal, 20 lung, 2 liver and 4 adrenal metastases. The median SBRT fraction and total dose were 6 Gy (6–10) and 30 Gy (30–50), respectively. The median number of fractions was 5 (3–5).

### 3.2. Treatment Outcomes

Treatment outcomes are summarized in Table 2.

The median follow-up was 24 months (range 6–42); 6–12–18-month LC rates were 100%, 70% and 41% respectively.

The median NEST-FS was 20 months (12–not reached). All pts had performed imaging control after a single course of SBRT: 53% experienced a CR, 35% a PR and 20% SD. No progressive disease was recorded. The ORR was 88% while the clinical benefit was 100%.

No > G2 adverse events related to SBRT were recorded.

## 4. Discussion

In mRCC progressing under first-line systemic treatment, the historical standard of care was based on the interruption of the current therapy and switching to a new line of treatment [14]. Historically, RCC has been considered a radioresistant tumor and RT was used only as palliative treatment, especially for brain and painful bone metastases. [15] In the last few years, with the employment of SBRT as a curative treatment to control oligoprogressive sites, radioresistance can be overcome and current therapy can be preserved [15,16].

In fact, according to the literature, it seems that SBRT overcomes the paradigm of the exclusively palliative role of RT in mRCC in favor of an active and safe treatment option that can be integrated into a multidisciplinary strategy to improve patients’ clinical outcomes.

To date, the results of the Delphi consensus on the use of SBRT in oligometastatic and oligoprogressive RCC divided expert opinion. However, 71% of panelists would recommend SABR as a valid option for delaying the change of systemic therapy in oligoprogressive disease in selected cases, and they suggest a minimum acceptable time of 6 months from the start of systemic therapy to SABR to avoid systemic treatment change [17]. Several studies showed that the addition of RT to systemic treatment with ICIs and TKIs induced cell death through the release of molecules known as damage-associated molecular patterns (DAMPs). Moreover, evidence also demonstrated that tumor cell populations with a significant survival advantage are not confined within the boundaries of an organ site but can successfully spread to and reseed other sites [17,18,19]. DAMPs make cancer cells more susceptible to a cytotoxic immune response, preventing metastasis-to-metastasis seeding [20,21,22]. Furthermore, the current literature has shown that SBRT on RCC metastases provides fair LC with acceptable toxicity [23,24].

Altoos et al. [23], after a 2-year follow-up, reported a LC rate of 93.4% with SBRT (vs. 35.27% with conventional RT) regarding 53 lesions in pts with extracranial disease. In this study, no pts reported severe adverse events. In a study conducted by Stinauer R.et al. [24], the univariate analysis showed a correlation between higher doses per fraction and LC in patients with mRCC. To our knowledge, all the retrospective studies in the literature investigated the role of SBRT in a heterogeneous series of pts who underwent different systemic therapies (chemotherapy, target therapy or immunotherapy).

Our study, instead, assessed the role of SBRT in patients treated with an ICIs–TKIs combination exclusively.

Our results showed that SBRT improved LC in pts treated with an ICIs–TKIs combination and the results seem to be similar to previous studies, although conducted on pts undergoing different types of therapies and without any significant toxicity.

As a coprimary endpoint, we chose NEST-FS. NEST-FS is an endpoint used to explore the activity and efficacy of SBRT in oligoprogressive disease to prolong current treatments and delay the change of systemic therapies. It was used especially in metastatic oligoprogressive prostate cancer.

To our knowledge, this endpoint has never been used in oligoprogressive mRCC.

Hannan et al., in a phase II clinical trial, showed that sequential SBRT to oligoprogressive mRCC metastases prolonged the duration of ongoing systemic therapy by >6 mo in at least 40% of patients [25].

Cheung et al., instead, showed that the use of SBRT, in oligoprogressive mRCC patients who had previous stability or response after 3 months of TKI therapy, guarantees a LC rate of 93% (95% CI 71–98%) at 1 year, with a median time to change in systemic therapy of 12.6 months [26].

Although our study included a very heterogeneous cohort of nodal, spine and visceral metastases, our results confirm that SBRT may provide good LC, with a 20-month median NEST-FS. Moreover, no severe acute and late toxicities were reported.

Nevertheless, the retrospective nature of these studies does not claim to obtain a strong level of evidence.

Therefore, further prospective studies are warranted to select patients who may benefit from SBRT in oligoprogressive mRCC and establish the optimal schedule of treatment to guarantee the best clinical oncological outcomes as possible.

## 5. Conclusions

SBRT upon oligoprogressive sites in mRCC treated with an ICIs–TKIs combination is an effective and feasible therapeutic strategy to overcome resistance to systemic treatment with a good local control and without significant toxicities. Further investigations are needed to obtain a strong level of evidence.

## Figures and Tables

**Table 1:** Patients characteristics.

*Variable*	n	%
**Age**		
≤61	22	65
>61	12	35
**Sex**		
Male	30	88
Female	4	12
**Performance status**		
0	20	59
1	10	29
2	4	12
**Metastatic sites undergone SBRT (56 metastases)**		
bone	14	41
lung	20	58
lymph node	16	47
liver	2	6
adrenal	4	11
**Imaging techniques**		
CT	30	88
MRI	14	41
**Systemic Therapy**		
axitinib-pembrolizumab	20	59
cabozantinib-nivolumab	14	40
**SBRT dose per fraction**		
30 in 6 Gy per fractions	30	54
37.5 Gy in 7.5 Gy per fractions	22	38
50 Gy in 10 Gy per fractions	3	4
40 Gy in 8 Gy per fractions	1	2

**Table 2:** Treatment outcomes.

OUTCOMES	
6 months-LC	100%
12 months-LC	70%
24 months-LC	41%
Median NEST	20 months
**SBRT RESPONSE RATE**	
CR	53%
PR	35%
SD	20%
ORR	88%
Clinical Benefits	100%

## Data Availability

No new data were created or analyzed in this study. Data sharing is not applicable to this paper.
